# Conditioning of pretreated birch by liquid–liquid organic extractions to improve yeast fermentability and enzymatic digestibility

**DOI:** 10.1039/d3ra02210b

**Published:** 2023-07-04

**Authors:** Guochao Wu, Björn Alriksson, Leif J. Jönsson

**Affiliations:** a Shandong Key Lab of Edible Mushroom Technology, School of Agriculture, Ludong University 264025 Yantai China; b Department of Chemistry, Umeå University SE-901 87 Umeå Sweden; c RISE Research Institutes of Sweden AB SE-891 22 Örnsköldsvik Sweden

## Abstract

By-products from hydrothermal pretreatment of lignocellulosic biomass inhibit enzymatic saccharification and microbial fermentation. Three long-chain organic extractants (Alamine 336, Aliquat 336 and Cyanex 921) were compared to two conventional organic solvents (ethyl acetate and xylene) with regard to conditioning of birch wood pretreatment liquid (BWPL) for improved fermentation and saccharification. In the fermentation experiments, extraction with Cyanex 921 resulted in the best ethanol yield, 0.34 ± 0.02 g g^−1^ on initial fermentable sugars. Extraction with xylene also resulted in a relatively high yield, 0.29 ± 0.02 g g^−1^, while cultures consisting of untreated BWPL and BWPL treated with the other extractants exhibited no ethanol formation. Aliquat 336 was most efficient with regard to removing by-products, but the residual Aliquat after the extraction was toxic to yeast cells. Enzymatic digestibility increased by 19–33% after extraction with the long-chain organic extractants. The investigation demonstrates that conditioning with long-chain organic extractants has the potential to relieve inhibition of both enzymes and microbes.

## Abstract 2

Long-chain extractants with suitable properties improve both saccharification and fermentation.

## Graphical abstract

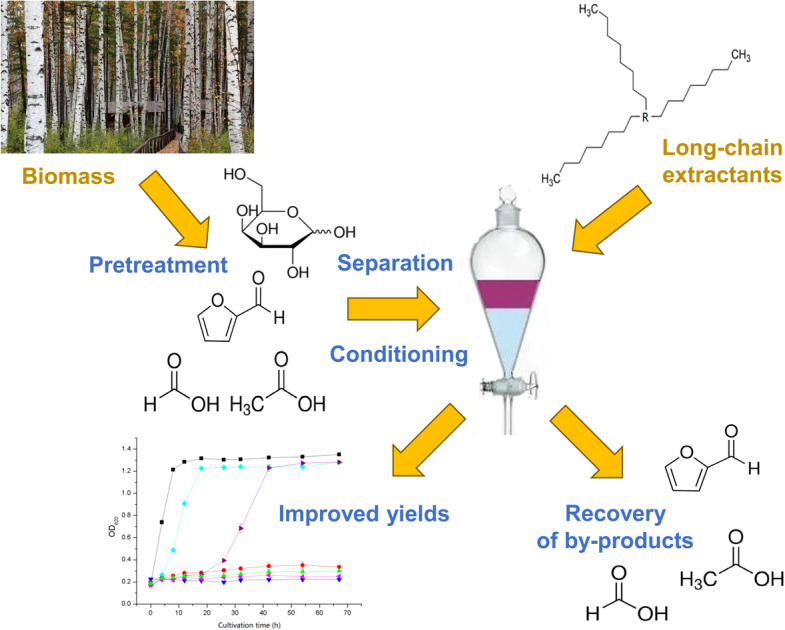

## 1 Introduction

Lignocellulosic residues from forestry and agriculture are abundant renewable resources for the production of advanced biofuels and other products that can be obtained through microbial fermentation processes.^1–3^ Prior to saccharification and fermentation, lignocellulose is subjected to pretreatment to make the cellulose more accessible to cellulolytic enzymes.^1–4^ There is a wide range of different pretreatment or fractionation methods.^1,2^ Hydrothermal pretreatment, with or without added acid catalyst and sometimes performed using steam explosion, is one of the most common approaches.^4^ Hydrothermal pretreatment primarily targets hemicelluloses, although cellulose and lignin are also affected.^4^ Hydrolysis lignin refers to the solid residue remaining after pretreatment, enzymatic saccharification, and microbial fermentation of sugars.^4^ The hydrolysis lignin can be used as an energy carrier or valorized to bio-based chemicals and materials.^5^

During pretreatment a wide range of inhibitors are generated together with the fermentable sugars.^6,7^ Microbial inhibitors include aliphatic acids, aliphatic aldehydes, benzoquinones, furan aldehydes, and phenylic compounds (*i.e.*, phenolic and non-phenolic aromatics).^8–10^ Inhibitors of enzymatic saccharification are less well characterized, but phenolic compounds and other aromatics seem to be important.^11–13^ This is further supported by the observation that hydrophilization of inhibitors is an important factor for relieving inhibition of enzymes.^7,14^

To alleviate problems with inhibitors, conditioning using alkali, including Ca(OH)_2_ (overliming), NaOH, and NH_4_OH, are commonly used.^6^ However, conditioning with alkali, such as overliming, can result in problems such as degradation of fermentable sugars and formation of precipitates.^15^ Therefore, conditioning methods other than overliming have been investigated, including treatment with reducing agents, ion exchange, enzymatic treatment with laccase and peroxidase, and liquid–liquid extraction (LLE).^6,7^ These methods have been found to be useful for alleviating inhibition of microorganisms, and in some cases also inhibition of cellulolytic enzymes. Several extractants, including ethyl acetate, Alamine, and supercritical CO_2_, have been studied with regard to treatment of lignocellulosic hydrolysates through LLE.^16–18^

**Fig. 1:**
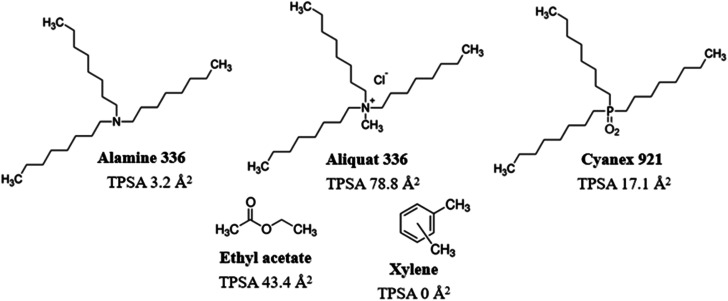
Structure and topological polar surface area (TPSA) of main components of the extractants.

Long-chain amines (such as Alamine 336), ammonium salts (such as Aliquat 336) and phosphine oxides (such as Cyanex 921) (Fig. 1) are important extractants used for the recovery of acids^19,20^ and for the removal of heavy metals.^21,22^ The main advantages of long-chain organic extractants include high equilibrium constants, high efficiency, low treatment temperatures, and low energy usage. LLE of corn stover prehydrolysates using mixtures with Alamine was shown to remove major fractions of acetic acid and furan aldehydes and improve ethanol production using the yeast *Pichia stipitis*.^16^ However, Aliquat extraction and Cyanex extraction have not yet been investigated with regard to conditioning of lignocellulosic hydrolysates for improved fermentability. Furthermore, potential effects of LLE on inhibition of cellulolytic enzymes are not well studied.

In this investigation, we compared the effects of extraction using Alamine 336, Aliquat 336 and Cyanex 921 on the liquid phase of pretreated birch (birch wood pretreatment liquid, BWPL). The three long-chain extractants share similar molecular structure in the sense that they all have three octyl groups, but they differ regarding the groups in the center, as Alamine 336 is a tertiary amine, Aliquat 336 is a quartenary ammonium salt, and Cyanex 921 is a phosphine oxide (Fig. 1). Two conventional solvents, ethyl acetate and xylene, were included for comparison. Ethyl acetate has previously been utilized for extraction of inhibitors from lignocellulosic hydrolysates and would therefore serve as a benchmark in studies of other extractants.^17,23^ Xylene differs from ethyl acetate in the sense that it is an aromatic substance with lower polarity.

The removal of inhibitors (including aliphatic acids, furan aldehydes, and total phenolics) from BWPL using all five extractants was investigated using chemical analysis. The effects of LLE of BWPL on enzymatic saccharification and fermentability using *Saccharomyces cerevisiae* yeast were also explored. Potential toxic effects of the five extractants on *S. cerevisiae* were also investigated. Investigations in this area can provide useful methods for making bioconversion of lignocellulosic residues to bio-based commodities more efficient, provide a better understanding of inhibition and conditioning phenomena, and result in new ways to isolate pretreatment by-products for further refining to co-products.

## 2 Results and discussion

### 2.1 Extraction of birch pretreatment liquid

Five extractants were used for detoxification of pre-treatment liquid from birch wood. The concentrations of sugars and common pretreatment by-products before and after extraction are shown in Table 1. The results indicate that none of the extractions affected the concentrations of the two main sugars in the pretreatment liquid, *i.e.*, xylose and glucose. Aliquat 336 was the most efficient extractant for all pretreatment by-products studied (Table 1). Aliphatic acids (acetic acid, formic acid, and levulinic acid) were reduced by 55%, furans (furfural and HMF) were reduced by 78%, and phenolics were reduced by 75%. With regard to aliphatic acids and total phenolics, the three long-chain organic extractants were more effective than ethyl acetate and xylene. The fraction of aliphatic acids removed by the long-chain organic extractants was 10–55%, whereas the corresponding fraction for ethyl acetate and xylene was only 5%. The fraction of total phenolics that was removed was 75% for Aliquat 336, 52% for Cyanex 921, 46% for Alamine 336, 28% for ethyl acetate, and 14% for xylene (calculated from data in Table 1). The removal of furan aldehydes followed a different pattern. Except for Aliquat 336, the other two long-chain organic extractants were less efficient than ethyl acetate and xylene (Table 1). The fraction of furan aldehydes that was removed was 78% for Aliquat 336, 72% for ethyl acetate, 58% for xylene, 56% for Cyanex 921, and 34% for Alamine 336.

Concentrations of sugars, aliphatic acids, furan aldehydes, total phenolics, total aromatic content and colour index in birch pretreatment liquid before (non-extracted) and after extraction with five extractants^a^

**Table:** 

Birch pretreatment liquid	Sugars		Aliphatic acids		
	Glucose (g L^−1^)	Xylose (g L^−1^)	Acetic acid (g L^−1^)	Formic acid (g L^−1^)	Levulinic acid (g L^−1^)
Non-extracted	12.64 ± 0.23	84.79 ± 1.59	20.56 ± 0.02	1.61 ± 0.03	0.11 ± 0.01
Alamine 336	11.76 ± 0.31	81.72 ± 2.08	15.45 ± 0.03	1.41 ± 0.03	0.09 ± 0.01
Aliquat 336	12.35 ± 0.09	82.72 ± 0.58	9.03 ± 0.02	0.93 ± 0.04	0.01 ± 0.01
Cyanex 921	12.16 ± 0.21	83.34 ± 1.58	18.97 ± 0.04	0.98 ± 0.04	0.03 ± 0.01
Ethyl acetate	12.64 ± 0.21	87.52 ± 1.54	21.15 ± 0.09	1.47 ± 0.09	0.12 ± 0.01
Xylene	11.99 ± 0.16	82.38 ± 1.21	20.79 ± 0.04	1.61 ± 0.01	0.11 ± 0.01

a Aliphatic acids refer to acetic acid, formic acid, and levulinic acid. Furan aldehydes refer to furfural and HMF.

b Total phenolics, indicated as vanillin equivalents, were determined using the Folin–Ciocalteu assay.

c TAC (total aromatic content) was measured at 280 nm using UV-VIS spectroscopy and pretreatment liquid diluted 200×.

d Colour index was measured at 465 nm using UV-VIS spectroscopy and pretreatment liquid diluted 20×.

**Table:** 

Birch pretreatment liquid	Furan aldehydes		Total phenolics^b^ (g L^−1^)	TAC^c^	Colour index^d^
	Furfural (g L^−1^)	HMF (g L^−1^)			
Non-extracted	3.57 ± 0.04	0.38 ± 0.01	8.96 ± 0.26	2.65 ± 0.06	0.37 ± 0.02
Alamine 336	2.31 ± 0.05	0.31 ± 0.01	4.84 ± 0.23	1.77 ± 0.03	0.08 ± 0.01
Aliquat 336	0.75 ± 0.02	0.12 ± 0.01	2.22 ± 0.11	0.64 ± 0.02	0.03 ± 0.01
Cyanex 921	1.48 ± 0.02	0.27 ± 0.01	4.31 ± 0.41	1.22 ± 0.07	0.07 ± 0.01
Ethyl acetate	0.93 ± 0.01	0.27 ± 0.01	6.99 ± 0.64	1.22 ± 0.04	0.29 ± 0.02
Xylene	1.32 ± 0.01	0.33 ± 0.01	7.73 ± 0.22	1.66 ± 0.01	0.38 ± 0.01

The total aromatic content (TAC) before and after extraction is shown in Table 1. All extractions removed a substantial part of TAC (33–76%). The fraction of TAC that was removed was 76% for Aliquat 336, 54% for Cyanex 921 and ethyl acetate, 37% for xylene, and 33% for Alamine 336 (calculated from data in Table 1). As both furan aldehydes and phenolics contribute to TAC, as well as non-phenolic aromatics, it is as expected that the pattern for removal of TAC did not exactly follow either any of the furan aldehydes or the total phenolics (Table 1).

Most of the extractions also decolourized the pretreatment liquid, as indicated by colour index measurements at 465 nm (Table 1). Extraction with xylene did not significantly change the colour index, and ethyl acetate was rather inefficient (<25% decrease, calculated from data in Table 1). In contrast, the three long-chain organic extractants decreased the colour index with >75%. The only similarity between TAC and colour index was that Aliquat 336 was the extractant that removed most absorbing substance.

**Fig. 2:**
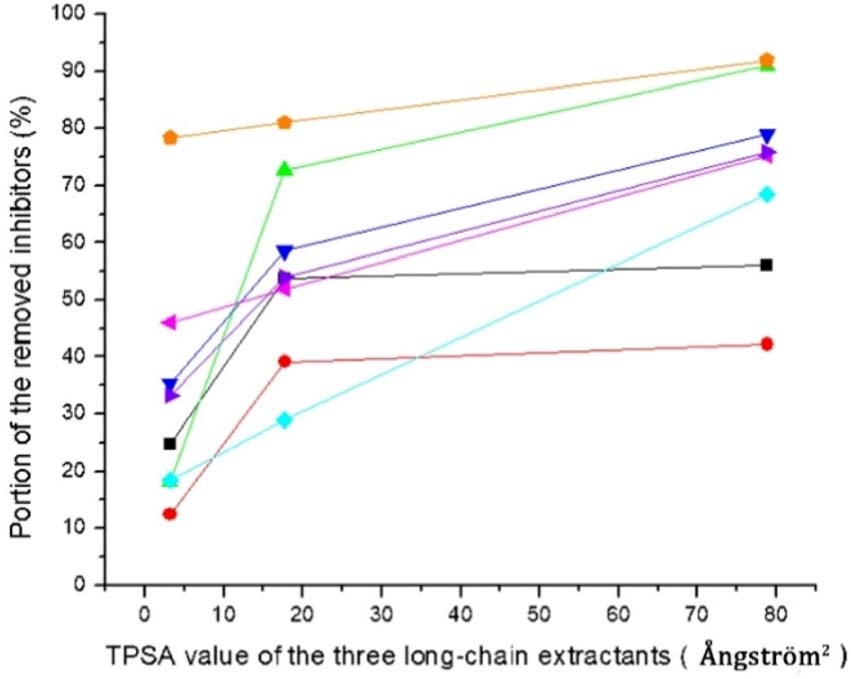
Removal of inhibitors in relation to the TPSA values of the three long-chain extractants. TPSA value: Alamine 336, 3.2 Å^2^; Cyanex 921, 17.7 Å^2^; Aliquat 336, 78.8 Å^2^. Acetic acid (■), formic acid (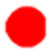), levulinic acid (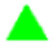), furfural (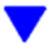), HMF (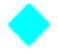), total phenolics (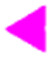), total aromatics (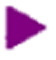) and colour index (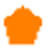).

The three long-chain extractants efficiently removed the inhibitors. Except for acetic acid, the efficiency of the three long-chain extractants in removing inhibitors increased with the increase of their topological polar surface areas (TPSA, Fig. 2). Removal of inhibitors and groups of inhibitors with hydroxyl groups, such as HMF and total phenolics, exhibited almost linear relationship with TPSA values (Fig. 2). Large topological polar surface area may enable better interactions between extractant and inhibitor, and thereby improve the extraction from the pretreatment liquid.

Jeong *et al.*^18^ showed that Alamine extraction reduced the concentration of glucose in a hydrolysate with only about 1%. Our results show that the small effect on sugar is also valid for other long-chain extractants, such as Aliquat 336 and Cyanex 921. Previous investigations suggested that extraction using Alamine 336 could partially remove inhibitors including furfural, HMF, and acetic acid from lignocellulosic hydrolysates or simulated hydrolysate.^16,18,24^ In our study, the total concentration of acetic acid and levulinic acid removed by Alamine 336 was 5.13 g L^−1^, which was higher than in the study by Jeong *et al.*^18^ However, the concentration of formic acid that was removed was lower in our study. The reason may be that the initial concentration of acetic acid in BWPL in our study was over five times higher than in the simulated hydrolysate used in the study by Jeong *et al.*,^18^ and extraction of acetic acid was the dominating event in the extraction of the chemically complex BWPL. Previous studies showed that furfural was removed more efficiently than 5-HMF using Alamine 336.^16,18^ Our results indicate that Aliquat 336 and Cyanex 921 were also able to remove furfural more efficiently.

### 2.2 Toxicity of extractants

**Fig. 3:**
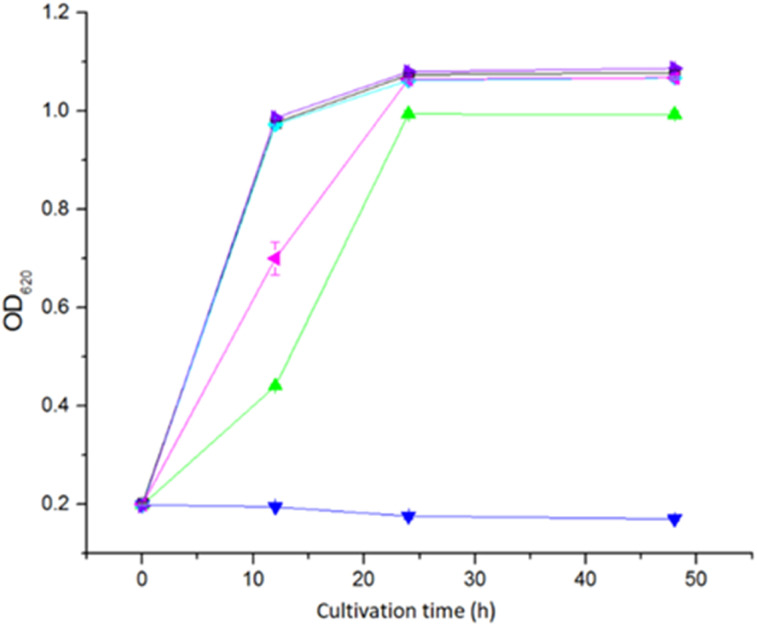
Toxicity test of the extractants on *Saccharomyces cerevisiae* Ethanol Red cells with microtiter plate assay. Every point in the graph was calculated as the mean value of three separate cultivations, and the error bars indicate standard deviations. Culture medium without extractant solution (■), Alamine 336 solution (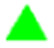), Aliquat 336 solution (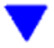), Cyanex 921 solution (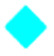), ethyl acetate solution (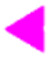), and xylene solution (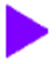). Lines and symbols of non-extracted medium, medium with Cyanex 921, and medium with xylene are overlapping and may therefore be difficult to discern.

The toxicity of the five extractants was tested in a microtiter plate assay with *S. cerevisiae* Ethanol Red yeast. Extraction with Cyanex 921 and xylene did not result in any toxic effect on yeast (Fig. 3). Extraction with ethyl acetate and Alamine 336 resulted in a weak toxic effect. For ethyl acetate extraction, the OD_620_ value was the same as for the non-extracted control after 24 h, whereas the OD_620_ value for Alamine 336 never reached the level of the non-extracted control (Fig. 3). Extraction with Aliquat 336 resulted in the most toxic effect, and no growth could be detected within the 48 h that the experiment lasted (Fig. 3).

The toxicity of extractants to microorganisms is a factor that must be considered when using LLE before fermentation processes. A previous investigation showed that Alamine 336 and Aliquat 336 were both toxic to lactic acid bacteria during extractive fermentation,^25^ and Aliquat 336 was more toxic than Alamine 336. Our study showed that Alamine 336 and Aliquat 336 were both toxic to *S. cerevisiae*, and Aliquat 336 showed higher toxicity.

Previous studies showed that the toxicity of Cyanex to the yeasts *Kluyveromyces marxianus* and *Pichia fermentans* was less than that of Alamine 336 when used for *in situ* extraction and recovery of metabolites from fermentation.^26^ In our study, Cyanex 921 did not inhibit the growth of *S. cerevisiae* cells.

The xylene-extracted water solution was not toxic to yeast cells (Fig. 3). That could be attributed to the low polarity (reflected by the TPSA value in Fig. 1) resulting in low solubility in the water phase.

Alamine 336, Aliquat 336 and Cyanex 921 have hydrophobic octyl chains, and could potentially accumulate in cell membranes. Toxic effects of Alamine 336 and Aliquat 336 on microorganisms may be related to accumulation in the cytoplasmic cell membrane, where extractants would disrupt the lipid bilayer and generate damage through metabolite leakage. The solubility of Cyanex 921 in water is low, and only low concentrations of residual Cyanex 921 would be left in the aqueous phase after extraction.^27^ The risk of Cyanex 921 causing membrane damage is thus low. However, Aliquat 336, which has the largest topological polar surface area (TPSA, Fig. 1), would be expected to have the highest water solubility among the three long-chain extractants. That may explain why Aliquat 336 showed the highest toxicity among the three long-chain extractants compared in this study.

### 2.3 Fermentation of extracted and non-extracted BWPL

**Fig. 4:**
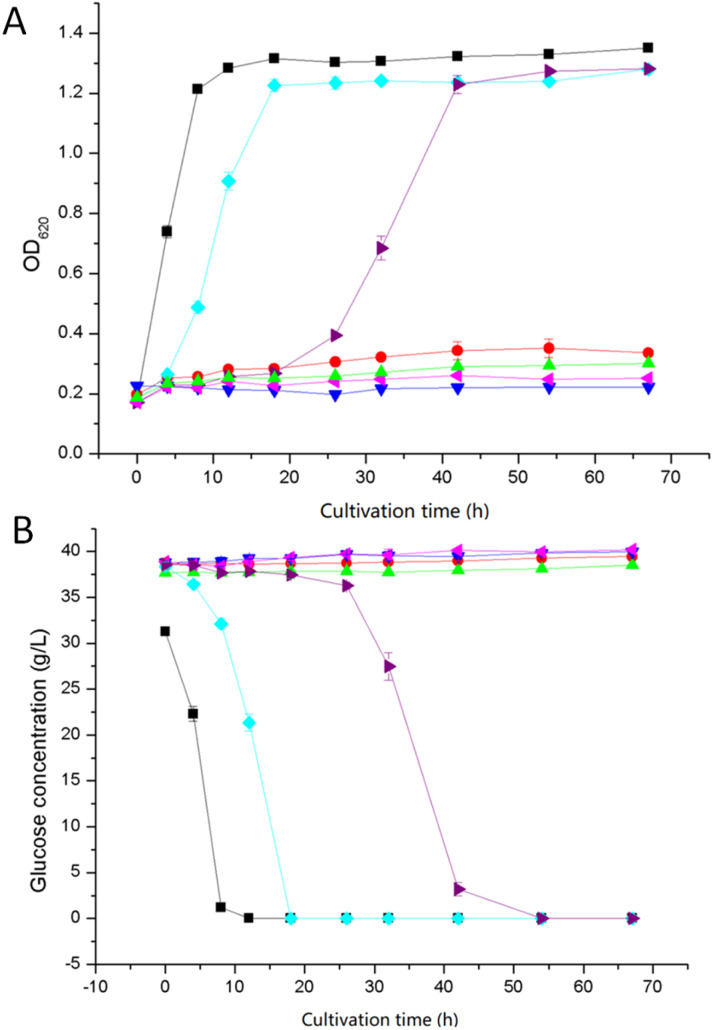
Fermentation of extracted and non-extracted BWPL with yeast. Every point in the graph was calculated as the mean value of two separate fermentations, and the error bars indicate standard deviations. Graphs show (A) yeast cell growth, and (B) glucose consumption. Culture medium without BWLP (■), non-extracted BWPL (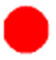), BWPL extracted with Alamine 336 (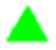), BWPL extracted with Aliquat 336 (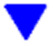), BWPL extracted with Cyanex 921 (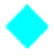), BWPL extracted with ethyl acetate (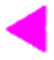), and BWPL extracted with xylene (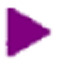).

Extracted and non-extracted BWPL was used in experiments with yeast and the effects of the extractions were evaluated by monitoring growth (as indicated by OD_620_) (Fig. 4A), glucose consumption (Fig. 4B), ethanol productivity after 18 and 42 h (Table 2), and ethanol yield on consumed hexose sugars (Table 2). As the Ethanol Red strain does not ferment xylose,^28^ the formation of ethanol should be strongly connected to glucose utilization, although BWPL might contain low concentrations of other potentially fermentable hexose sugars, such as mannose and galactose.

**Table:** Enzymatic hydrolysis and ethanolic fermentation with a BWPL-less reference and BWPL before and after extractions^e^

Medium	Glucose yield^a^ (g L^−1^)	*Q*_18 h_ b (g L^−1^ h^−1^)	*Q*_42 h_ b (g L^−1^ h^−1^)	*Y*_E/G_ c (g g^−1^)
Reference	47.4 ± 2.1	0.64 ± 0.02	0.11 ± 0.01	0.37 ± 0.02
Non-extracted BWPL	20.6 ± 0.6	NDTD^d^	NDTD	NDTD
Alamine 336/BWPL	25.1 ± 0.7*	NDTD	NDTD	NDTD
Aliquat 336/BWPL	27.7 ± 0.3*	NDTD	NDTD	NDTD
Cyanex 921/BWPL	24.8 ± 0.8*	0.86 ± 0.03*	0.16 ± 0.01*	0.34 ± 0.02*
Ethyl acetate/BWPL	15.5 ± 0.9	NDTD	NDTD	NDTD
Xylene/BWPL	23.9 ± 0.8	NDTD	0.15 ± 0.01*	0.29 ± 0.02*

a Glucose yield based on the results after 45 h of enzymatic hydrolysis of 5% Avicel in the presence or absence of birch pretreatment liquid.

b Volumetric ethanol productivity based on the results within the first 18 h or 42 h of fermentation.

c *Y*_E/G_ is the ethanol yield on the initial amount of fermentable sugar (galactose, glucose and mannose). The calculations are based on the maximum ethanol concentrations obtained within 67 h of fermentation.

d NDTD, none detected.

e *values which were significantly larger (*P* < 0.05 with Student's *t*-test) than those with the non-extracted BWPL.

There was very little growth when yeast was cultivated with non-extracted BWPL (Fig. 4A). This agrees with non-extracted BWPL exhibiting no or little glucose consumption (Fig. 4B) and ethanol formation (Table 2). Under the conditions used, 40% BWPL was sufficiently toxic to entirely inhibit yeast. Extractions of BWPL using Cyanex 921 and xylene were the only treatments that resulted in clear improvement of growth (Fig. 4A), consumption of glucose (Fig. 4B), and ethanol formation after 42 h (Table 2). Extraction with Cyanex 921 was superior compared to extraction with xylene, as judged from shorter lag phases for growth and glucose consumption (Fig. 4), ethanol formation already after 18 h (Table 2), and slightly better values for *Q*_42 h_ and *Y*_E/G_ (Table 2). When using xylene-extracted BWPL, yeast started to grow after a lag phase of about 18 h (Fig. 4A). The volumetric productivities for Cyanex-921-extracted BWPL after 18 and 42 h and for xylene-extracted BWPL after 42 h were higher than the corresponding values for the reference fermentation without BWPL (Table 2). This can be explained by higher glucose concentration in BWPL-containing medium (as indicated by the zero values in Fig. 4B) and small amounts of other hexose sugars than glucose in the BWPL. The initial concentrations of galactose in BWPL extracted with Cyanex 921 and xylene were 3.43 and 3.28 g L^−1^, respectively. The corresponding values for mannose were 7.84 g L^−1^ for Cyanex 921 and 7.83 g L^−1^ for xylene. Thus, both media contained around 11 g L^−1^ of galactose and mannose. The results were conclusive also with regard to extraction with Alamine 336, Aliquat 336, and ethyl acetate: no growth, no glucose consumption, and no ethanol production.

The inhibitory effects of pretreatment liquids from wood would normally be expected to be more severe than those from pretreatment liquids from agricultural residues, as the pretreatment conditions used for wood are typically harsher.^7^ Therefore, pretreated wood usually needs a more thorough detoxification. In the current study, the relatively high concentrations of inhibitors left in BWPL after extraction may be related to the high initial concentrations. As a result, the extracted BWPLs still exhibited toxicity.

Ethyl acetate was used for treatment of steam-exploded poplar wood, and the treatment resulted in some improvement of the ethanol yield, which reached 51% of the theoretical value.^17^ Our results showed that ethyl acetate extracted some inhibitors of ethanolic fermentation. However, the BWPL used in our study was still very toxic, and the removal of the inhibitors was not sufficient for improving the fermentability.

Despite that only small fractions of inhibitors were removed by the extraction, xylene improved the fermentability of BWPL. The reason might be that xylene extracted inhibitors that were present in low concentrations, but which had high molar toxicity. As reported previously, the molecular toxicity of inhibitors is very different, and some of the most toxic inhibitors are phenolics.^29,30^ They are relatively lipophilic and tend to be extracted by xylene.

### 2.4 Effects of extraction on enzymatic saccharification of cellulose

Table 2 shows the glucose yields from enzymatic saccharification of Avicel in media consisting of non-extracted and extracted BWPL and, as a control, in media without BWPL. The glucose formation in the presence of non-extracted BWPL was only 44% of the glucose formation in the control without BWPL. Some inhibition will be caused by end-product inhibition by sugars in the BWPL. Hydrophilic substances, such as sugars, will not be affected by solvent extraction, as can be seen in Table 1. However, solvent extraction might remove more lipophilic inhibitors, such as aromatic substances, and thereby improve saccharification in reaction mixtures with extracted BWPL compared to saccharification in reaction mixtures with non-extracted BWPL.

Except for extraction with ethyl acetate, saccharification of reaction mixtures with extracted BWPL was improved with 16–33% compared to reaction mixtures with non-extracted BWPL (Table 2). The increase in glucose formation after extraction was 21% for Alamine 336, 33% for Aliquat 336, and 19% for Cyanex 921. All of these increases were statistically significant (Student's *t*-test, *P* < 0.05). Extraction with xylene increased glucose production with 16%. The ethyl-acetate-extracted BWPL was even more inhibitory than non-extracted BWPL. Thus, the only extractants that had a positive effect on both fermentability and saccharification were Cyanex 921 and xylene.

Apart from feedback inhibition by sugars, inhibition of cellulases is mainly caused by aromatic substances in the pretreatment liquid.^7,11–13,31^ Our results show that Aliquat 336, which performed best with respect to removal of total aromatics, also improved enzymatic saccharification most efficiently. Detoxification through addition of sulphur oxyanions, which react with some aromatic compounds but not with sugars, is often efficient for conditioning of slurries/hydrolysates.^14,32,33^ As shown by the decrease of total phenolics and TAC (Table 1), the LLE method removes aromatic inhibitors through extraction. Regarding the decrease in released sugar using ethyl-acetate-extracted BWPL, our results agree with an attempt to detoxify steam-exploded poplar wood using ethyl acetate.^17^ In that study, the glucose concentration produced after extraction with ethyl acetate was even lower than for hydrolysis of crude biomass.^17^ The effect can be attributed to the lower polarity of furfural compared to 5-HMF.

Zhu *et al.*^16^ removed 73% acetic acid and over 45% furan aldehydes from corn stover prehydrolysate using mixed extraction with Alamine, *n*-octanol, and kerosen. The concentrations of acetic acid and furan aldehydes in that study were 3.74 g L^−1^ and 0.17 g L^−1^, respectively, and were thus much lower than the corresponding concentrations in our study (20.56 g L^−1^ acetic acid and 3.95 g L^−1^ furan aldehydes, Table 1).

It has been reported that ethyl acetate removes acids, furan aldehydes and phenols.^17,23^ In our study, all types of inhibitors except for acetic acid could be removed by ethyl acetate. Aghazadeh *et al.*^23^ used vacuum evaporation to remove residual ethyl acetate due to the high residual ethyl acetate after the extraction. Vacuum evaporation was not applied in our study, and the initial concentration of acetic acid was larger in our study than in that of Aghazadeh *et al.*^23^ These reasons can have contributed to the lack of effect on acetic acid in our study.

It was not surprising that xylene did not extract much carboxylic acids, as the polarities of xylene and carboxylic acids differ significantly. However, our results showed that xylene was favourable for extraction of furfural produced from lignocellulosic biomass, which agrees with a previous study.^34^ Notably, xylene did not extract as much phenolics as Aliquat 336, which has a relatively polar center (Fig. 1). That might be due to the relatively high polarity of some abundant phenolics present in the pretreatment liquid, such as vanillin and ferulic acid.^6,26^

Persson *et al.*^35^ used supercritical fluid to extract inhibitors from a lignocellulosic hydrolysate of spruce and improve the subsequent ethanol fermentation. The method was effective with regard to removal of furfural, leaving only 7% furfural in the hydrolysate after extraction.^35^ Compared to the supercritical fluid extraction, the three long-chain extractants used in our study were advantageous with respect to removal of carboxylic acids. The surfactants L62D and L62LF, which consist of both hydrophilic and hydrophobic parts, have also been used in detoxification of lignocellulosic hydrolysate.^36^ While the surfactants efficiently removed more lipophilic inhibitors,^36^ such as phenolics, the long-chain extractants used in the current study were capable of removing both lipophilic and hydrophilic inhibitors, including carboxylic acids, furans and phenolics.

## 3 Experimental

### 3.1 Pretreatment

The birch wood pretreatment liquid (BWPL) used in this study was produced from European white birch (*Betula pubescens*). The pretreatment of birch was performed by SEKAB E-Technology AB using a 30 L pretreatment reactor in the Biorefinery Demo Plant (Örnsköldsvik, Sweden). Birch wood chips were pretreated at 190 °C (16 bar over-pressure) with sulfur dioxide as catalyst.

The feed rate was 14 kg h^−1^ (dry weight) and the wood chips were impregnated with sulfur dioxide at a rate of 0.7 kg h^−1^. The residence time was 7 min. After pretreatment, the slurry, which had a pH of 1.8 and dry-matter content of 12%, was cooled and stored at 4 °C until further use.

### 3.2 Extraction of the pretreatment liquid

Alamine 336 (trioctylamine), Aliquat 336 (trioctylmethyl-ammonium chloride), Cyanex 921 (trioctylphosphine oxide), ethyl acetate, and xylene were used as extractants. In 100 mL Erlenmeyer flasks, 30 mL of BWPL (with the pH maintained at 1.8) was mixed with 10 mL of extractant. The flasks were shaken at 180 rpm and 25 °C for 30 min. After extraction, the mixtures were transferred to a separatory funnel for separation, and the aqueous lower phase (the extracted BWPL) was collected for further experiments.

### 3.3 Effects of extractants on *S. cerevisiae*

A set of experiments was performed with 2% (w/v) glucose-based medium to investigate potential toxic effects of the extractants on *S. cerevisiae* (Ethanol Red, Fermentis Ltd, Marcq-en-Baroeul, France) in the absence of BWPL. The yeast inoculum was prepared in a 50 mL Falcon tube with YPD medium (2% yeast extract, 1% peptone, 2% d-glucose). The Falcon tube was inoculated with freeze-dried yeast and incubated with agitation at 30 °C for approximately 4 h. The cells were harvested by centrifugation at 1500*g* for 5 min. The cells were then resuspended in an appropriate volume of sterilized deionized water to give an inoculum with a final biomass concentration of 2 g L^−1^ DW.

Deionized water was thoroughly mixed with each extractant (with an aqueous phase : organic phase ratio of 3 : 1). The aqueous phase was then separated from the organic phase using a separation funnel and collected by pipetting.

Triplicate experiments were performed in 96-well microtiter plates. Each well in the microtiter plates was filled with 285 μL glucose-based medium, or, alternatively, with medium consisting of the aqueous phases obtained after extraction with 2% (w/v) glucose added. Each well was supplemented with 6 μL of a nutrient solution (150 g L^−1^ yeast extract, 75 g L^−1^ (NH_4_)_2_HPO_4_, 3.75 g L^−1^ MgSO_4_·7H_2_O, 238.2 g L^−1^ NaH_2_PO_4_·H_2_O), and 9 μL of yeast inoculum. The final volume in each well was 300 μL out of the maximum 330 μL.

Airproof adhesive film was used to seal the microtiter plates to avoid well-to-well contamination and sample evaporation, and to keep an oxygen-limited environment. Control wells filled with only culture medium without inoculum were included to confirm there was no cross contamination. The plate was then incubated at 30 °C in a shaker incubator (Ecotron, Infors AG, Bottmingen, Switzerland) set at 180 rpm. The OD (optical density) at 620 nm was measured after 0, 12, 24, and 48 h using a Victor2 1420 Multilabel Counter (PerkinElmer, Waltham, MA, USA).

### 3.4 Fermentation of BWPL

Fermentation experiments were conducted to assess potential detoxification effects of the extractions when using *S. cerevisiae* Ethanol Red yeast. The fermentations were performed in serum flasks with 25 mL medium. The flasks were equipped with magnets for stirring and were sealed with rubber plugs pierced with cannulas for release of carbon dioxide. The pH of the BWPL and the extracted BWPLs was adjusted to 5.5 using a 5 M aqueous solution of sodium hydroxide. The flasks were filled with 23.75 mL diluted BWPL (the final fraction of BWPL was 40%, and the total volume was 25 mL), detoxified BWPL (final fraction 40%) or sterile ultrapure water (the reference). Glucose was added to each flask to a final concentration of 30 g L^−1^ glucose. Each flask was also supplemented with 0.5 mL of the nutrient solution (Section 3.3), and 0.75 mL of yeast inoculum. The yeast inoculum was prepared as described in Section 3.3 and the size of the inoculum was 2 g L^−1^ DW in all fermentation vessels. The experiments were done in duplicates. The flasks were incubated at 30 °C in a water bath with multi-point magnetic stirring (IKA-Werke, Staufen, Germany). Samples for measurement of sugars and ethanol were withdrawn after 0, 4, 8, 12, 18, 26, 32, 42, 54, and 67 h.

### 3.5 Effects on enzymatic saccharification of cellulose

Experiments with BWPL and extracted BWPL were performed to investigate if the extraction would affect enzymatic saccharification of cellulose. The pH of the BWPL and the BWPL from the extraction experiments was adjusted to 5.2 using a 5 M aqueous solution of sodium hydroxide. The mixture contained BWPL (final fraction 50%), sodium citrate buffer (50 mM, pH 5.2), 50 mg Avicel PH-101 (microcrystalline cellulose obtained from Sigma-Aldrich), and 20 mg of an enzyme cocktail consisting of equal proportions of the two liquid enzyme preparations Celluclast 1.5 L (15 FPU g^−1^) and Novozym 188 (30 BU g^−1^), both of which were obtained from Sigma-Aldrich. The total mass of the reaction mixtures was 1000 mg. References with 50 mM sodium citrate buffer (pH 5.2) and without any BWPL were also included. Experiments were performed in triplicate.

The reaction mixtures were incubated for 45 h at 45 °C in an orbital shaker (Ecotron incubator shaker) set at 170 rpm. Samples for measurement of sugars were withdrawn at 0 and 45 h. Glucose levels in saccharification experiments were initially followed by using a glucometer (Accu-Chek Active, Roche Diagnostics, Basel, Switzerland). To calibrate the glucometer, a standard curve was prepared using a series of glucose solutions with known concentrations. Before measurements, saccharification samples were diluted 30 times. The concentration values were recalculated using the standard curve. To obtain more accurate values, the glucose levels were afterwards determined using HPAEC, as described in Section 3.6. All reported values come from the HPAEC analyses.

### 3.6 Analyses

All samples were diluted with ultra-pure water and filtered through 0.2 μm nylon membrane filters (Millipore). Analysis of glucose, xylose, acetic acid, formic acid, and levulinic acid was performed with HPAEC (high-performance anion-exchange chromatography) using an ICS-5000 instrument (Dionex, Sunnyvale, CA, USA) equipped with an electrochemical detector, a CarboPac PA1 (4 × 250 mm) separation column, and a CarboPac PA1 (4 × 50 mm) guard column (all from Dionex). The temperature of the column oven was 30 °C. Prior to injection, the column was regenerated with a solution of 260 mM sodium hydroxide (Sodium Hydroxide Solution for IC, Sigma-Aldrich) and 68 mM sodium acetate (Anhydrous Sodium Acetate for IC, Sigma-Aldrich) during 12 min followed by ultra-pure water during 2 min. Each sample was injected once, and elution was performed with ultra-pure water during 25 min.

Analysis of furfural and 5-hydroxymethylfurfural (HMF) was performed by using high-performance liquid chromatography (HPLC). A Zorbax RRHD SB C18 column (Agilent) was used in an Agilent Technologies 1200 series HPLC system. The device was equipped with an autosampler, a refractive index detector (RID), a binary pump, and a degasser (all from the Agilent 1200 series).

The total phenolic contents in the pretreatment liquids were estimated using the Folin–Ciocalteu method. A mixture was prepared of 10 μL properly diluted sample, 150 μL of a 20% (w/w) aqueous solution of Na_2_CO_3_, 700 μL deionized water, and 50 μL Folin–Ciocalteu reagent (Sigma-Aldrich). The mixture was allowed to react for 2 h at room temperature (22 °C) before measuring the absorbance at 760 nm. Vanillin was used as the standard.

The total aromatic content, which includes heteroaromatics, such as 5-hydroxymethylfurfural (HMF) and furfural, and aromatics, such as phenols, was measured as absorbance at 280 nm using a UV1800 spectrophotometer (Shimadzu, Kyoto, Japan). This wavelength was selected as quantitatively important aromatics and heteroaromatics have absorption maxima close to 280 nm (vanillin, 279 nm; HMF, 284 nm; furfural, 278 nm). The colour index was measured as absorbance at 465 nm using the UV1800 spectrophotometer.

The production of ethanol was determined off-line using the Agilent Technologies 1200 series HPLC system. The chromatographic separation was performed using an Aminex HPX-87H column (Bio-Rad Laboratories AB, Solna, Sweden). Separation was achieved with an isocratic gradient of 0.01 N H_2_SO_4_ at a flow-rate of 0.6 mL min^−1^ and the temperature of the column oven was set to 60 °C. The temperature of the detector cell of the RID was set to 55 °C. An external calibration approach was applied for quantification.

## 4 Conclusions

In this study, we investigated for the first time the conditioning of a lignocellulosic hydrolysate with Aliquat 336 and Cyanex 921, and compared these two extractants with Alamine 336, ethyl acetate, and xylene. This investigation shows that detoxification of hydrolysates using LLE can promote not only fermentability, but also enzymatic saccharification. Aliquat 336 was most effective with regard to removal of inhibitors, but residual Aliquat 336 left in the pretreatment liquid after extraction was toxic to yeast. Improved fermentation with yeast was observed for extractions with Cyanex 921 and xylene. Extractions with Alamine 336, Aliquat 336, and Cyanex 921 significantly improved enzymatic saccharification, and Aliquat 336 was most effective. As extraction with Cyanex 921 and xylene promoted both yeast fermentation and enzymatic saccharification they appear most attractive for industrial use. The investigation also revealed that the efficiency of the three long-chain extractants with respect to inhibitor removal increased with increasing topological polar surface area (TPSA). This may be helpful for finding novel and efficient long-chain extractants in the future. Detoxification using LLE can be performed at room temperature and at acidic pH, which contributes to making it practical from a technical point of view. Importantly, there was almost no sugar loss during the extraction. Recovery of extracted acids, furans and phenolics, and recycling of extractants warrant further investigation in the future.

## Author contributions

Guochao Wu: formal analysis, investigation, validation, visualization, writing – original draft. Björn Alriksson: conceptualization, resources, writing – review & editing. Leif Jönsson: conceptualization, funding acquisition, project administration, resources, supervision, writing – review & editing.

## Conflicts of interest

BA and LJ are inventors of patents in the area biomass processing.

## Supplementary Material
